# Editorial: Digital Innovation and Data-Driven Research in Neurodegenerative Diseases

**DOI:** 10.3389/fneur.2022.961847

**Published:** 2022-06-30

**Authors:** Wei Gu, Panying Rong, Martin Hofmann-Apitius, Venkata Satagopam

**Affiliations:** ^1^Luxembourg Centre for Systems Biomedicine, University of Luxembourg, Esch-sur-Alzette, Luxembourg; ^2^Department of Speech-Language-Hearing: Sciences & Disorders, The University of Kansas, Lawrence, KS, United States; ^3^Fraunhofer Institute for Algorithms and Scientific Computing, Fraunhofer Gesellschaft, Sankt Augustin, Germany

**Keywords:** digital innovation (DI), data driven, wearable devices, sensor, neurodegenerative diseases

Interdisciplinary research efforts, especially those involving digital innovation and data-driven research have emerged to aid in the development of novel assessment tools for neurodegenerative diseases. With the rapid development, especially in applying innovative machine learning and biomedical signal processing techniques, these novel assessment tools can provide a more efficacious means of evaluating the disease and treatment-related physical and mental changes, thus facilitating the discovery of new treatments to improve the quality of life and survival for patients with neurodegenerative diseases.

In this Research Topic, we have published several high quality research works covering Research Topics from “information flow pattern,” “emergency room evaluation,” “digital technology for individualized treatment,” to “detection of cognitive impairment.”

In the work of He et al., the brain information flow pattern in patients with early mild cognitive impairment (EMCI) has been defined using non-parametric multiplicative regression-Granger causality analysis on MRI images and neurological measures data. The identified disturbed pattern in EMCI patients compared to healthy controls shows a promising application in supporting the diagnosis of EMCI.

Beauchet et al. have developed a tool for Emergency Room Evaluation and Recommendations (ER^2^). They have screened for older Emergency Department (ED) visitors at high risk of undesirable events and examine the performance criteria of the ER^2^ high-risk level and its “temporal disorientation” item alone to screen for major neurocognitive disorders in older ED visitors. The resulting work suggested a potential method to screen for major neurocognitive disorders in older ED users. This will accelerate the diagnosis of those targeted patients and so that better care can be provided at a timely manner.

Fröhlich et al. have systematically reviewed the current developments and advancements in digital technology toward a better individualized treatment of Parkinson's Disease. They started the review by summarizing and commenting on recent efforts related to digital biomarkers in Parkinson's Disease, including digital biomarkers for speech and voice impairments, hypomimia, Gait impairment, impairment of handwriting, and those derived from other biometric data modalities. Then they addressed the topic of data science and its role as an enabler of digital biomarkers. They further provided an outlook of the emerging future of digital biomarkers in precision neurology and discussed the ethical, legal, and social aspects of digital biomarker research and its application in individualized medicine.

Ambadi et al. presented their work on detection of cognitive impairment using the Spatio-Semantic Graphs (SSG) from picture description from more than 1,300 audio recordings. They have shown the ability to identify pre-clinical cognitive changes using this method and discussed its limitations. This pilot work can be used as a guidance for future larger scale studies.

Though substantial progress has been made, using data from the medical field is still facing many challenges. The most notable one is the lack of high quality and standardized data, especially for the training of machine learning models, which requires a large amount of data. For example, the Electronic Health Records (EHR) data are often unstructured, some even not digitized. The works reported in this Research Topic mostly have to undergo data curation before the analysis and model building. Implementation of standards at the data entry/collection step could substantially reduce the downstream data curation and harmonization efforts.

The sensor and mobile devices and apps arena is still wild west at this moment, data coming from these devices are rathor specific to the vendors. In some cases raw data is not provided for research. It is crucial to define and/or adopt standards for the outcome to facilitate cross device data analysis. Some efforts have been invested in this direction, such as the Michael J. Fox Foundation's digital health strategy emphasizing mobile-generated data standards development.

While wearable and mobile devices enable continuous activity monitoring and recording in uncontrolled, real-world environments, generating high-dimensional digital health data, how to extract biologically and clinically relevant information from these data remains an ongoing challenge. To address this challenge, collaborative efforts among engineers, biomedical scientists, data scientists, and clinicians are needed to continuously develop and refine analytical approaches and measurement techniques to maximize the potential of digital health data.

We urge the research community to devote more work in solving the data quality challenge and share curated data. This is the key to accelerate digital innovation and data-driven research and unlock its great potential in real world applications.

In addition, it is very important to provide medical informatics training to physicians and physician-scientists to prepare them for and engage them in the future development such as new decision making tools and other data driven technologies.

With the current technological advancements, we are witnessing the beginning of data-driven digital-health changes in the medical landscape. However, we should always bear in mind that all innovations and technology developments should respect the privacy of the users. ELSI requirements should be met to earn trust of the society so that technologies could at the end contribute to individualized precision medicine in reality.

The new paradigm of digital readouts has already been addressed by the Innovative Medicines Initiative (IMI) and other EU funding initiatives. Projects such as RADAR-CNS (https://www.radar-cns.org), RADAR-AD (https://www.radar-ad.org) and Mobilize-D (https://www.mobilise-d.eu/) have paved the way for a wide adoption of digital readouts and digital endpoints in studies focusing on neurodegenerative diseases. The successor of IMI, the Innovative Health Initiative (IHI) will certainly build on the portfolio developed in the course of IMI. What is required in the future are projects that combine digital readouts and molecular biomarker readouts, in order to establish a rational basis for the replacement of questionnaires and cognitive test batteries by digital “day-to-day” recordings. In the long run, we will see large cohorts being characterized solely by digital recordings using wearables and handheld devices; a “trial-ready” cohort of the future will be a cohort characterized and qualified through digital readouts.

## Author Contributions

WG and VS wrote the first draft of the manuscript. PR and MH-A wrote sections of the manuscript. All authors contributed to manuscript revision, read, and approved the submitted version.

## Conflict of Interest

The authors declare that the research was conducted in the absence of any commercial or financial relationships that could be construed as a potential conflict of interest.

## Publisher's Note

All claims expressed in this article are solely those of the authors and do not necessarily represent those of their affiliated organizations, or those of the publisher, the editors and the reviewers. Any product that may be evaluated in this article, or claim that may be made by its manufacturer, is not guaranteed or endorsed by the publisher.

